# Hemophagocytic Macrophages Harbor Salmonella enterica during Persistent Infection

**DOI:** 10.1371/journal.ppat.0030193

**Published:** 2007-12-14

**Authors:** Rebecca N Nix, Sarah E Altschuler, Peter M Henson, Corrella S Detweiler

**Affiliations:** 1 Department of Molecular, Cellular, and Developmental Biology, University of Colorado, Boulder, Colorado, United States of America; 2 Department of Pediatrics, National Jewish Medical and Research Center, Denver, Colorado, United States of America; 3 Department of Immunology, National Jewish Medical and Research Center, Denver, Colorado, United States of America; Tufts University School of Medicine, United States of America

## Abstract

Salmonella enterica subspecies can establish persistent, systemic infections in mammals, including human typhoid fever. Persistent S. enterica disease is characterized by an initial acute infection that develops into an asymptomatic chronic infection. During both the acute and persistent stages, the bacteria generally reside within professional phagocytes, usually macrophages. It is unclear how salmonellae can survive within macrophages, cells that evolved, in part, to destroy pathogens. Evidence is presented that during the establishment of persistent murine infection, macrophages that contain S. enterica serotype Typhimurium are hemophagocytic. Hemophagocytic macrophages are characterized by the ingestion of non-apoptotic cells of the hematopoietic lineage and are a clinical marker of typhoid fever as well as certain other infectious and genetic diseases. Cell culture assays were developed to evaluate bacterial survival in hemophagocytic macrophages. *S.* Typhimurium preferentially replicated in macrophages that pre-phagocytosed viable cells, but the bacteria were killed in macrophages that pre-phagocytosed beads or dead cells. These data suggest that during persistent infection hemophagocytic macrophages may provide *S.* Typhimurium with a survival niche.

## Author Summary

Microbes that establish persistent infections present serious problems for world health but are not well understood. The bacteria Salmonella enterica cause asymptomatic chronic infection in humans. Carriers shed the bacteria into the environment, leading to periodic acute typhoid fever epidemics. Antibiotics are effective at treating typhoid fever, but Salmonellae strains resistant to multiple antibiotics have caused recent epidemics. New therapeutic strategies are needed and may develop from a molecular understanding of how the bacteria avoid killing by our immune systems. During acute and chronic infection, Salmonellae reside within macrophages, a kind of white blood cell type that normally destroys bacteria. Evidence is presented that during the establishment of chronic infection of mice, the bacteria can live within a special kind of macrophage. Hemophagocytic macrophages are macrophages that have ingested white and red blood cells. They are a clinical marker of typhoid fever and many other kinds of microbial infections. Cell culture assays showed that Salmonellae preferentially survive in hemophagocytic macrophages. These data suggest that hemophagocytic macrophages may provide *S.* Typhimurium with a survival niche during chronic infection. Moreover, a natural mouse model and a cell culture assay now exist for studying the medically important phenomenon of hemophagocytosis.

## Introduction

Salmonella enterica are Gram-negative bacteria that are acquired from contaminated food or water. Certain S. enterica subspecies can traverse the gut lumen of some mammals and then colonize lymphatic tissue, causing systemic infection. S. enterica subspecies Typhi colonize the human liver, spleen, and mesenteric lymph nodes, causing Typhoid fever. Approximately 5% of people with acute Typhoid fever progress to an asymptomatic chronic infection. These individuals intermittently shed the pathogen into community sewers and thereby serve as a reservoir for dissemination to naïve hosts [1]. Little is known about how bacteria establish chronic infections in otherwise healthy mammals.

S. enterica subspecies Typhimurium cause infections of the liver, spleen, and mesenteric lymph nodes in mice. Like humans, mice can develop acute infections that progress to chronic infections. Historically, researchers have focused on the acute phase of infection using mouse strains that are homozygous for a loss of function mutation in the vacuolar cation transporter Slc11a1 (Nramp1). Slc11a1^G169D^ mutant mice serve as a good model for acute infection because they are exquisitely sensitive to intravacuolar eukaryotic and bacterial pathogens [2]. For instance, they generally die within a week of inoculation with virulent *S.* Typhimurium. In contrast, Slc11a1 wild-type mice infected with *S.* Typhimurium survive acute infection and develop chronic infections that last for months or longer [3,4]. In this report we exploit Slc11a1 wild-type mice to investigate how *S.* Typhimurium establish chronic infection.

To determine where *S.* Typhimurium reside during the early stages of chronic infection, we examined tissue sections from orally inoculated Slc11a1 wild-type mice. The bacteria were found within macrophages that had ingested other cell types. Macrophages that have ingested other cell types are also known as hemophagocytic macrophages. *S.* Typhimurium infection of hemophagocytic macrophages was modeled using primary mouse macrophages and a macrophage-like tissue culture cell line. Data suggest that *S.* Typhimurium survive and replicate within macrophages that phagocytosed viable host cells but are killed by macrophages that phagocytosed nothing or that phagocytosed dead host cells. These results indicate that hemophagocytic macrophages may provide *S.* Typhimurium with a survival niche in vivo during persistent infection.

## Results

### *S.* Typhimurium-Infected Tissues Contain Macrophages That Have Phagocytosed Other Blood Cell Types

**Figure 1:**
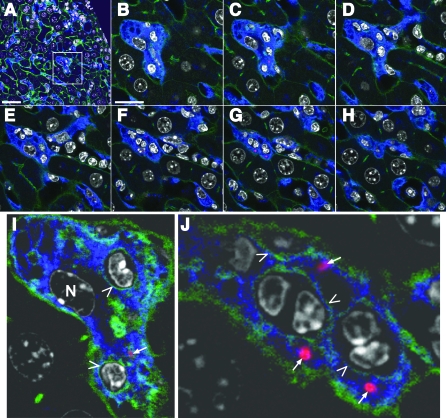
*S.* Typhimurium Reside in Macrophages That Appear to Have Multiple Nuclei Confocal fluorescence microscopy of 50-μm-thick liver sections from a 1-wk-infected Slc11a1 (Nramp1) wild-type mouse. *S.* Typhimurium (O-antigen, arrows) are red, macrophages (F4–80 and MOMA-2) are blue, DNA (DAPI) is gray, and phalloidin is green. (A) Low power image, scale bar is 40 μm. (B–H) Montage of 4-μm optical sections through the boxed region of (A). Scale bar is 20 μm. (I and J) Enlarged images showing actin rings (arrowheads) around nuclei within the multinucleate macrophage. The endogenous macrophage nucleus is visible in (I) and is labeled with an N. The video from which (A–J) were derived (Video S1) is available online.

**Figure 2:**
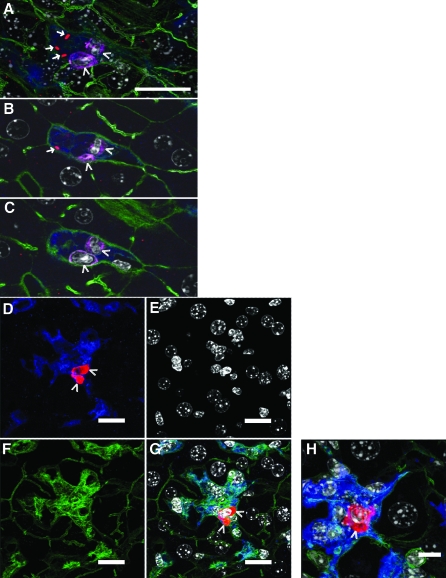
*S.* Typhimurium-Infected Macrophages Containing Phagocytosed Neutrophils and T Cells Confocal fluorescence microscopy of 50-μm-thick liver sections from 1-wk-infected Slc11a1 wild-type mice. (A–C) *S.* Typhimurium (O-antigen, arrows) are red, macrophages (F4–80 and MOMA-2) are blue, DNA (DAPI) is gray, phalloidin is green, and neutrophils (Gr-1/Ly-6G/RB6-8C5) are pink (arrowheads). (A) Collapsed image from a 40-μm Z-stack. Scale bar is 20 μm. (B and C) Sections from (A) that are 4 μm apart. The video from which (A–C) were derived (Video S2) is available online. (D–G) T cells within multinucleate macrophages. Macrophages (F4–80 and MOMA-2) are blue (D, G, and H), T cells (CD3_zeta_) are red (D, G, arrowheads), DAPI is gray (E, G), actin-bound phalloidin is green (F, G). (G) Is a composite of (D, E, and F). Scale bars are 16 μm. (H) An image from a different mouse stained and labeled as described for (D–G). Scale bar is 8 μm. A video showing a T cell inside of a macrophage is available online (Video S3).

To gain insight into how acute infections can become persistent infections, we examined the known sites of infection, livers, spleens, and mesenteric lymph nodes, in *S.* Typhimurium-infected 129SvEv Slc11a1 wild-type mice at 1-, 3- and 8-weeks post-infection. This time span represents several disease stages, including a period of strong innate immune response (1-week), strong adaptive immune response (3-weeks), and the beginning of clearance in some animals (8-weeks) [3,5–7]. Mice were inoculated intragastrically with 5 × 10^8^ virulent bacteria, a dose at which most individuals survive and become colonized for months or longer [4]. This resulted in approximately 10^4^ bacterial colony-forming units (CFU) per gram of spleen or liver at 1- and 3-weeks post-infection, and 10^3^ CFU per gram at 8-weeks post-infection. Fixed tissue sections were processed for immunofluorescence microscopy. *S.* Typhimurium was found within macrophages at all time points, consistent with previous reports [4,8,9]. Phalloidin labeling of actin facilitated visualization of tissue architecture and cell boundaries by confocal microscopy (Figures 1 and 2, Videos S1 and S2). The macrophages within the livers and spleens of infected mice appeared multinucleate at 1-, 3-, and 8-weeks post-infection. Multinucleate macrophages were not observed in mock-infected mice, indicating that they are a distinct feature of infection. Quantification of the bacteria within mouse tissues was difficult due to the low number of *S.* Typhimurium in persistently infected mice. For instance, previous researchers were routinely able to detect *S.* Typhimurium in acutely infected mice with thick-section confocal microscopy at bacterial loads of 10^5^ CFU/gram of tissue but could not visualize bacteria at 10^4^ CFU per gram of tissue [9]. The bacterial load in our mice, which had not undergone pre-selection for high levels of colonization [4], was 10^4^ CFU per gram of tissue. A confocal microscope with an acousto beam splitter enabled limited quantification of bacteria at this level of colonization. Data indicate indicated that most *S.* Typhimurium that were clearly intracellular were within multinucleate macrophages at 1- and 3- weeks post-infection (Table 1). At 8-weeks post-infection, there were too few visible bacteria (10^3^ CFU/gram of tissue) per 50 μm section to quantify, but the occasional bacteria we did find were within multinucleate macrophages (data not shown).

**Table 1:**
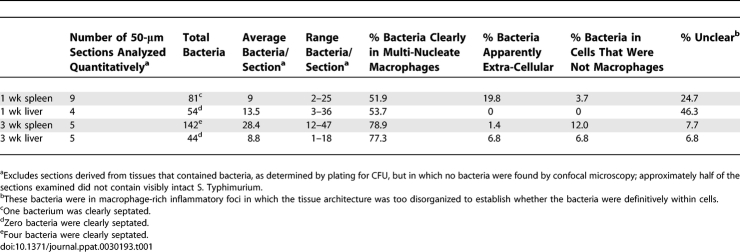
Quantification of Bacteria in Mouse Tissues

Several observations suggest that macrophages within infected tissues were multinucleate due, at least in part, to phagocytosis of other host cells. First, actin rings were observed around many of the nuclei (Figure 1I and 1J), consistent with phagocytosis [10,11]. Second, confocal microscopy with cell-type specific markers indicated that the nuclei represented cells of diverse types. Some of the nuclei likely represented engulfed macrophages, as the area immediately around them but within an actin ring was recognized with the macrophage-specific antibodies F4–80 (cell surface) and MOMA-2 (cytoplasmic) (data not shown). Other nuclei co-localized with a marker that recognizes neutrophils, specifically an antibody to Ly-6G/Gr-1, which stains peripheral granulocytes, including neutrophils (Figure 2A–2C) [12]. This suggests that some of the nuclei within macrophages were derived from phagocytosed neutrophils, which are normally recruited to sites of bacterial infection [9]. Additionally, hemophagocytic macrophages contained lymphocytes, as evidenced by staining with T or B cell specific markers (Figure 2D–2H, Video S3, and data not shown). The results collectively suggest that macrophages in *S.* Typhimurium-infected mice engulf multiple types of leukocytes and that such macrophages could provide *S.* Typhimurium with an in vivo niche.

### The Cells Phagocytosed by Macrophages in *S.* Typhimurium-Infected Tissues Appear to Remain Intact

**Figure 3:**
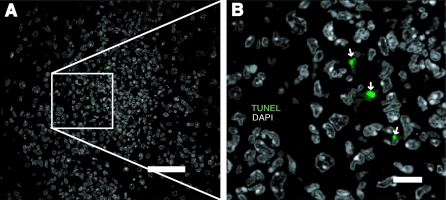
Infected Tissues Contain Few Terminal Deoxynucleotidyl Transferase (TdT)-Positive Nuclei Nick-end (TdT) labeling (green, arrows) and DAPI staining (gray) of a liver inflammatory lesion, 1 wk post-infection. (A) Scale bar is 40 μm. (B) Close-up of box in (A). Scale bar is 8 μm.

**Figure 4:**
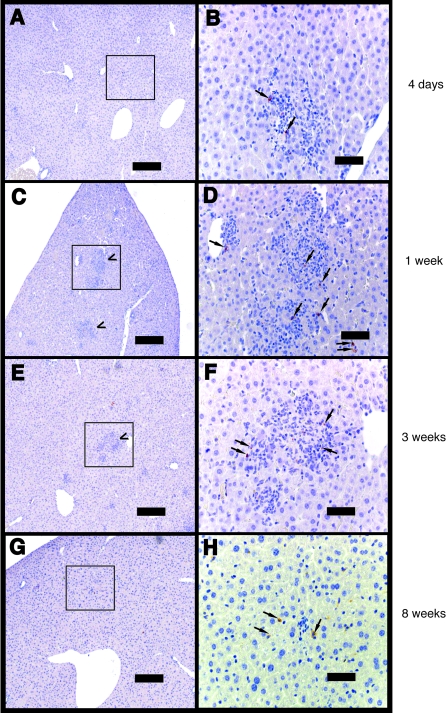
Infected Tissues Have Significant Inflammatory Lesions at 1 wk and 3 wk but Contain Few Caspase-3-Positive Cells Light microscopy of hematoxylin (purple), eosin (pink), and caspase-3 (brown, arrows) stained liver thin sections over an infection time course. Enlarged images of the boxed regions on the left (200-μm scale bars) are shown on the right (50-μm scale bars). Arrowheads show inflammatory lesions: (A–B) 4 d post-infection; (C–D) 1 wk; (E–F) 3 wk; and (G–H) 8 wk.

A normal function of tissue macrophages is to phagocytose and destroy dead or dying cells. However, several observations suggest that the engulfed host cells were not degraded at the time of tissue fixation. First, cell surface markers for different leukocytes were sufficiently intact to be detected (Figure 2). Second, the leukocyte nuclei within macrophages of *S.* Typhimurium infected mice appeared intact (Figures 1 and 2) even though nuclear fragmentation is a known indicator of cell death. A highly sensitive method of detecting broken DNA, nick-end labeling, revealed few damaged nuclei within inflammatory lesions at 1- and 3-weeks post-infection (Figure 3 and data not shown). Since nuclear and DNA fragmentation are late-stage markers of cell death, it was possible that the leukocytes ingested by macrophages in infected mice were at an earlier stage of death upon fixation. Tissue sections were examined for the presence of mature caspase-3, which can be detected prior to and after DNA breakage in apoptotic cells [13]. Few caspase-3 positive cells were seen in liver sections at 4-days or at 1-, 3-, or 8-weeks post-infection. During this time frame, small diffuse inflammatory lesions (4-days) developed into larger dense lesions (1- and 3-weeks) and finally resolved into small lesions (8-weeks) (Figure 4). However, significant death or degradation of the engulfed cells in infected tissues was not observed. Collectively, these observations suggest that ingested cells may have been alive upon phagocytosis and/or were not degraded by the hemophagocytic macrophages.

### *S.* Typhimurium Preferentially Survive in Activated Primary Mouse Macrophages That Have Phagocytosed Viable Leukocytes

**Figure 5:**
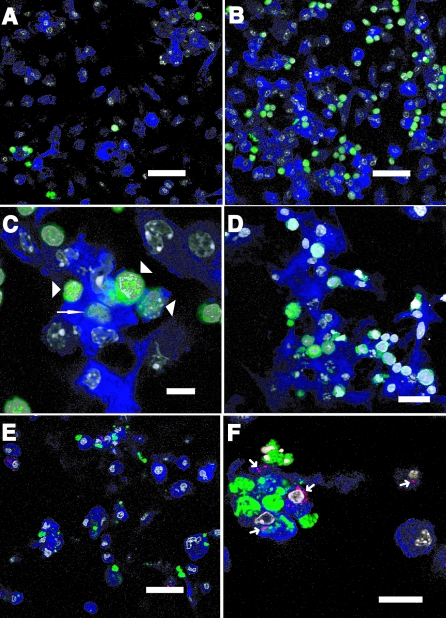
Activated Bone Marrow–Derived Macrophages (BMDMs) Phagocytose Live Host Cells Confocal fluorescence microscopy. BMDMs are blue (F4–80 and MOMA-2), human T cells (Jurkats) are green (CMFDA-stained), and DNA is gray (DAPI). (A–C) 30 min after the addition of Jurkats to BMDMs. (A) Unactivated BMDMs show little association with Jurkat cells, many of which were washed away. Scale bar is 40 μm (B and C). Activated macrophages phagocytose Jurkat cells; arrow denotes engulfed cell, arrowheads show partially engulfed cells. Scale bars are 40 μm (B) and 8 μm (C). (D–F) 42 h after mock-infection (D), or *S.* Typhimurium-infection (O-antigen, red, arrows, E and F). Scale bars are 20 μm (D), 40 μm (E), and 16 μm (F).

**Figure 6:**
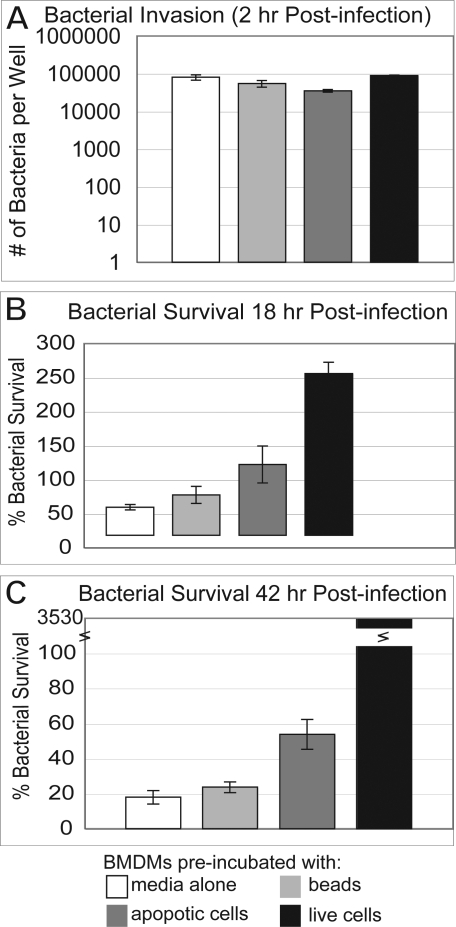
*S.* Typhimurium Preferentially Survive within BMDMs That Phagocytosed Live Host Cells Gentamicin protection assay analyzed by plating for colony-forming units (CFU). BMDMs were incubated with media alone (white bars), beads (light gray bars), apoptotic (dark gray bars), or live (black bars) cells (Jurkats) and then infected with *S.* Typhimurium. (A) Bacterial invasion, as determined 2 h post-infection. (B–C) *S.* Typhimurium survival expressed as percent of invasion (A). (B) 18 h post-infection and (C) 42 h post-infection.

To establish whether *S.* Typhimurium could preferentially survive within macrophages that have ingested viable versus dead host cells, an in vitro tissue culture infection assay was developed. Primary bone marrow–derived mouse macrophages (BMDMs) were generated from Slc11a1 wild-type mice. BMDMs were activated with the cytokine interferon-gamma (IFNγ) and LPS on the premise that in vivo *S.* Typhimurium are likely to encounter activated macrophages after the first few days of infection [14,15]. Activated BMDMs were incubated with media, polystyrene beads, apoptotic cells, necrotic cells (data not shown), or live cells. Within thirty minutes, both beads and cells were phagocytosed by the activated BMDMs (Figure 5B). As expected, many of the beads or cells added to the unactivated BMDMs were not phagocytosed and were therefore removed with washing (Figure 5A). *S.* Typhimurium was added to the BMDMs 1-hour after the addition of beads or cells. Thirty minutes later, gentamicin was added to kill extracellular bacteria. Two hours post-infection, intracellular *S.* Typhimurium were enumerated by plating lysed BMDMs on selective media. There were up to 2-fold differences in the number of bacteria in BMDMs across samples (Figure 6A), but the patterns of these differences varied between experiments and were not considered significant. By 18-hours post-infection, the number of intracellular *S.* Typhimurium declined in activated BMDMs that were pre-incubated with media only, beads, or dead cells (Figure 6B). This is consistent with previous observations that activated BMDMs effectively kill *S.* Typhimurium [16–18]. However, BMDMs that phagocytosed viable cells prior to infection exhibited 2-fold bacterial replication by 18-hours and 35-fold replication by 42-hours (Figure 6B and 6C). Similar results were obtained when BMDMs were incubated with Jurkat E6–1 cells, a human T cell derived line (Figure 6), or with DG-75 cells, a human B cell derived line (data not shown). These results indicate that *S.* Typhimurium survives and replicates preferentially within BMDMs that have phagocytosed viable cells.

**Figure 7:**
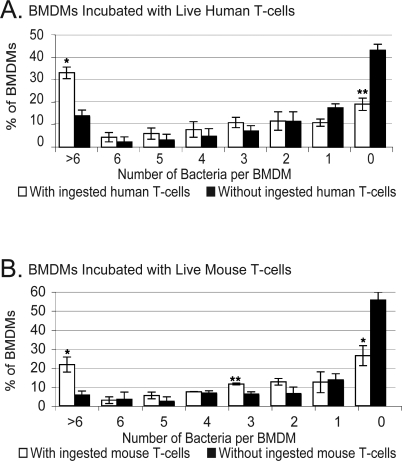
*S.* Typhimurium Preferentially Survive within Individual BMDMs That Phagocytosed Live Cells Gentamicin protection assays analyzed by fluorescence microscopy. Histograms of activated BMDMs that did (white bars) or did not (black bars) phagocytose live T cells. BMDMs were subsequently infected with *S.* Typhimurium. The *x*-axis indicates the number of bacteria (>6, 6, 5, 4, 3, 2, 1, or 0) per BMDM at 42 h post-infection. Experiments were performed with human (A) or mouse T cells (B). Student's *t*-test *p*-values are shown (**p < 0.01, * *p* < 0.05).

The plating assay described above is a population assay. To determine the status of bacterial replication in individual BMDMs upon infection with *S.* Typhimurium, the number of bacteria per BMDM was determined by immunofluorescence confocal microscopy. BMDMs were incubated with live host cells and scored based on whether or not live cells had been phagocytosed. The number of intracellular *S.* Typhimurium rods within each BMDM was enumerated. By 18-hours (data not shown) and 42-hours post-infection, BMDMs that had ingested viable human (Figures 5E, 5F, and 7A) or mouse (Figure 7B) T cells contained more bacteria than BMDMs on the same cover-slip that had ingested nothing. The observation that both mouse and human T-lymphocyte derived tissue culture cells have similar effects suggests that this phenomenon is not species specific. These results corroborate the colony-forming unit analyses (Figure 6) and indicate that macrophages which have phagocytosed viable cells could provide *S.* Typhimurium with a niche for replication.

### *S.* Typhimurium Preferentially Survive in Activated Mouse Macrophage-Like Tissue Culture Cells That Have Phagocytosed Viable Leukocytes

**Figure 8:**
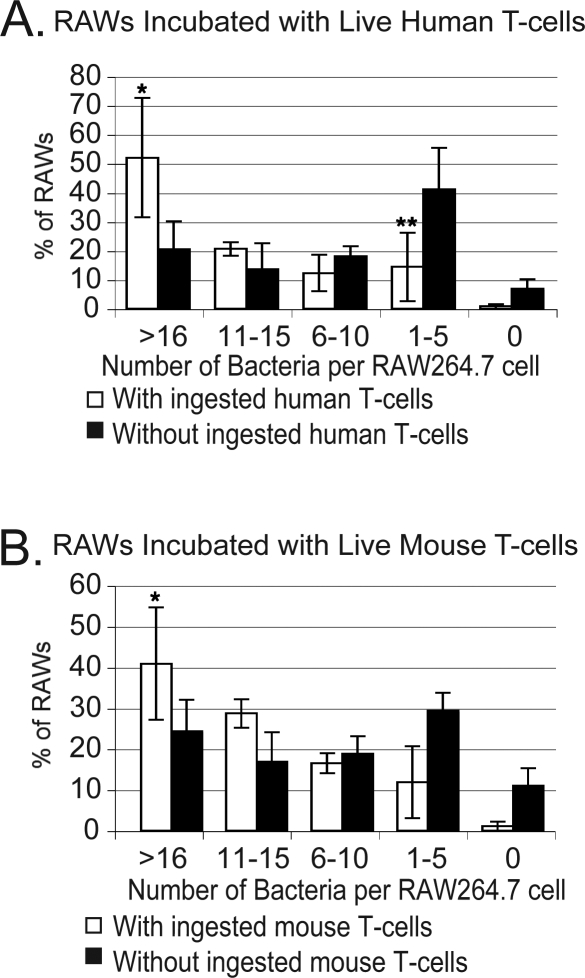
*S.* Typhimurium Preferentially Survive within Mouse Macrophage-Like Tissue Culture Cells That Phagocytosed Live Cells Gentamicin protection assays analyzed by fluorescence microscopy. Histograms of activated RAW264.7 (Slc11a1^G169D^) cells that did (white bars) or did not (black bars) phagocytose live T cells. RAW264.7 cells were subsequently infected with *S.* Typhimurium. The *x*-axis indicates the number of bacteria (>16, 11–15, 6–10, 1–5, or 0) per RAW264.7 at 42 h post-infection. Experiments were performed with human (A) or mouse T cells (B). Student's *t* test *p-*values are shown (**p < 0.01, * *p* < 0.05).

The BMDMs used above were derived from Slc11a1 wild-type mice. Many researchers work with Slc11a1^G169D^ (homozygous loss-of-function) mouse macrophage-like cell lines, such as J774s or RAW264.7s. These cells allow *S.* Typhimurium to replicate to much higher levels than their wild-type counterparts [19]. *S.* Typhimurium replication was compared in RAW264.7 cells that did, or did not, phagocytose viable leukocytes. RAW264.7 cells were activated with IFNγ, incubated with viable human or mouse T-lymphocyte derived tissue culture cells, and then infected with *S.* Typhimurium. Intracellular bacteria were enumerated as described above, but consistent results were not obtained. Observation of individual cells by fluorescence microscopy indicated that relative to activated BMDMs, fewer activated RAW264.7 cells phagocytosed live T cells; human and mouse T cells were engulfed by 52 ± 3% and 87 ± 5%, respectively, of BMDMs, compared to only 8 ± 2% and 68 ± 2%, respectively, of RAW264.7 cells. To determine whether individual RAW264.7 cells that did ingest viable T cells were permissive for *S.* Typhimurium replication, intracellular bacteria were enumerated using fluorescence microscopy 18-hours (data not shown) and 42-hours post-infection. Experimental variation was minimized by comparing RAW264.7 cells with or without ingested T cells from the same infection-wells. As expected, RAW264.7 cells were quite permissive for *S.* Typhimurium replication and multiple bacteria were enumerated per cell. Nevertheless, uptake of either human or mouse T cells correlated with increased bacterial load (Figure 8). This suggests that *S.* Typhimurium survival in macrophages that have phagocytosed viable cells is a phenomenon that can occur in cell lines as well as in primary cells and is Slc11a1-independent.

## Discussion

Hemophagocytosis is the phenomenon of activated macrophages engulfing cells of the hematopoietic lineage [20]. It can occur in patients infected with evolutionarily diverse microbial agents, including Staphylococci, Mycobacteriae, Leishmaniae, Epstein-Barr virus, and influenza virus [20,21]. Hemophagocytosis is also associated with genetic, neoplastic, and rheumatic disorders. Patients with hemophagocytosis typically experience fever, splenomegaly, and cytopenias, specifically leukopenia, anemia, and thrombocytopenia [20]. Bone marrow, liver, or spleen biopsies may reveal numerous hemophagocytic macrophages in these patients [21]. While it is clear that in clinical situations hemophagocytosis is pathological [20], it is unknown whether the phenomenon could benefit the host in certain situations or at sub-clinical levels.

Hemophagocytosis is an established clinical feature of human typhoid fever. English-language observations of hemophagocytosis in typhoid patients date back to 1898 with the description of large phagocytic cells containing red and white blood cells in livers obtained from patients who died during the first couple weeks of infection [22]. More recent papers also describe hemophagocytosis in typhoid patients, sometimes referring to the hyperphagocytic macrophages as “typhoidal cells” [23–29]. For example, in one study, bone marrow biopsies were performed on 40 juvenile patients who tested positive for typhoid, paratyphi A or paratyphi B by blood culture and agglutination. Thirty-four patients (85%) had macrophages that contained multiple cell types, including granulocytes, lymphocytes, blood platelets, and erythrocytes [30]. Thus, hemophagocytosis occurs in a significant subset of typhoid patients.

We observed hemophagocytosis in a mouse model of typhoid fever. Tissue sections from infected mice revealed macrophages with multiple nuclei (Figure 1). Many of the nuclei represented phagocytosed leukocytes, as indicated by the staining of material around these nuclei with cell-surface markers for neutrophils, T cells (Figure 2) and B cells (data not shown). It seems unlikely that the phenomenon observed is macrophage ingestion of dead leukocytes, as the leukocyte nuclei, DNA, and cell surfaces appeared intact. Moreover, infected tissues did not contain significant numbers of cells with activated caspase-3 (Figures 1–4). Based on these data, we hypothesized that infected tissues contained macrophages that had phagocytosed viable leukocytes, indicating that they were hemophagocytic.

It is difficult to experimentally determine within an animal model whether engulfed cells of infected tissues were alive or dead upon phagocytosis. Therefore a cell culture assay was developed to test the corollary that *S.* Typhimurium survives preferentially in macrophages that phagocytosed viable leukocytes versus dead or no leukocytes. This assay relied upon activating macrophages with the inflammatory cytokine interferon-gamma (IFNγ), which can play a major role in maintaining and possibly establishing hemophagocytosis in humans [31]. Severe systemic hemophagocytosis is often rapidly fatal due to a dramatic reduction in circulating red blood cells. Patients with severe hemophagocytosis have high IFNγ blood serum levels and can be successfully treated with a combination of inhibitory anti-IFNγ antibodies and blood transfusion. This indicates that IFNγ is important for maintenance of the pathological state, likely via macrophage activation [21]. One consequence of in vitro macrophage activation with IFNγ is increased phagocytosis of particles, including beads, dead cells, and live cells (Figure 5 and data not shown). Therefore, IFNγ-activated tissue culture macrophages that had engulfed different particles were used to establish whether macrophages that ingested viable host cells preferentially allow *S.* Typhimurium to survive. First, activated primary bone marrow–derived macrophages were incubated with beads, viable lymphocytes, or dead lymphocytes, each of which became phagocytosed (Figures 5 and 6). The macrophages were infected with *S.* Typhimurium and bacterial survival was evaluated over time. Bacteria were not visualized within lymphocytes under our experimental conditions. This is consistent with a report that *S.* Typhimurium does not infect T cells [32], but another group found that under some conditions, the bacteria can infect B cells [33]. In our experiments, *S.* Typhimurium survived and replicated only in macrophages that ingested viable cells (Figures 5–7). This was not likely a function of the number of phagocytosed live versus dead cells because similar numbers of each were engulfed by macrophages across experiments (Figure 6A). Analogous experiments were performed with RAW264.7 cells, a commonly used macrophage-like mouse cell line. Individual RAW264.7 cells that had phagocytosed viable mouse or human T-lymphocytes contained more intracellular *S.* Typhimurium than RAW264.7 cells that did not engulf T cells (Figure 8). These observations suggest that tissue culture macrophages as well as primary macrophages will be useful for identifying molecular mechanisms of *S.* Typhimurium replication in macrophages that engulfed viable cells. Moreover, *S*. Typhimurium survival in macrophages that engulfed viable mouse or human cells suggests that the phenomenon is not limited to mice. Thus, macrophage phagocytosis of viable cells and/or subsequent infection with *S.* Typhimurium may inhibit *S.* Typhimurium killing by the macrophage and could provide the bacteria with a survival niche in vivo*.*

It is surprising that the observation that *S.* Typhimurium can reside within hemophagocytic macrophages in mice has not been previously reported, particularly since immunofluorescence microscopy of tissue sections from *S.* Typhimurium infected mice has been performed by multiple laboratories [8,9,34]. One explanation for this discrepancy may be that salmonellae researchers historically work with mouse strains that are homozygous for Slc11a1^G169D^, and significant hemophagocytosis may not occur in these mice (e.g. C57Black6 and Balb/c strains). In light of this, it is interesting that activated Slc11a1^G169D^ RAW264.7 cells that phagocytosed viable lymphocytes do allow *S.* Typhimurium to replicate (Figure 8). This could suggest that the apparent absence of significant hemophagocytosis in Slc11a1^G169D^ mice is due to differences between the mutant and wild-type mice at the tissue and/or organismal level. Slc11a1^G169D^ mice experience abnormally high levels of bacterial replication in macrophages [9] and B cells [33], massive inflammatory infiltration into infected tissues, and death within a week [35]. It is possible that inflammation or death masks or prevents hemophagocytosis in these animals. Wild-type mice survive acute infection and become carriers. At 11 weeks post-infection, *S.* Typhimurium were found within macrophages in wild-type mice, but multinucleate macrophages or hemophagocytosis were not reported [4]. We observed bacteria in multinucleate macrophages as late as 8-weeks post-infection (data not shown), but have not examined tissues from later time points. One possibility is that hemophagocytic macrophages may represent a niche for *S.* Typhimurium survival early, but not late, during infection. This is of interest because salmonellae survival in hemophagocytic macrophages could play a role in the establishment of chronic infection. Future experiments will be needed to resolve these issues.

How might hemophagocytosis lead to the alteration of a macrophage such that it can no longer effectively control *S.* Typhimurium? One possibility is based on observations that macrophage interactions with viable cells involve receptor-ligand responses that can alter macrophage activation states [36,37]. Surface proteins on viable cells activate SIRPα/SHPS-1 on macrophages. SIRPα/SHPS-1 activation initiates an inhibitory tyrosine phosphatase cascade that blocks FcγR- and CR3-mediated phagocytosis, which are functional markers of macrophage activation [38,39]. Viable cell surface proteins that activate SIRPα/SHPS-1 include immunoglobulin superfamily member CD47/IAP and integrins [40]. These data suggest that viable cell activation of SIRPα and/or other macrophage receptors could partially or wholly inactivate macrophages such that they cannot control *S.* Typhimurium replication. Activation of surface receptors could also condition macrophages such that they are vulnerable to inactivation by an *S.* Typhimurium-specific mechanism. This is intriguing in part because there is evidence that systemic salmonellae can delay and/or blunt immune responses [41]. For instance, Salmonella enterica serotype Typhi has a capsule, Vi-antigen, that down-regulates the host TLR response, a major arm of the innate immune system [42,43]. Finally, it is also possible that cell surface receptor-ligand interactions alone are insufficient to alternatively-activate or condition macrophages, and that the actual engulfment of viable cells is required.

Additional evidence that macrophage activation states are altered upon interaction with large, live particles is found upon examination of NCBI GEO DNA microarray datasets. There are currently no datasets that examine macrophage gene expression changes upon exposure to viable leukocytes. However, analyses that quantify macrophage and dendritic cell responses to the single-celled eukaryotic pathogens Leishmania donovani, *Leishmania major,* and Toxoplasma gondii reveal multiple changes in genes that regulate or are markers of macrophage activation state, such as EST2, STX11, LST1, and HLA-DMB (see Table S1). This is consistent with the idea that the response of macrophages to interaction with other live eukaryotic cells is complex and can involve changes in activation state.

The in vivo and in vitro evidence suggest that *S.* Typhimurium may use hemophagocytic macrophages as a survival niche in mice, and that this phenomenon may model a clinical feature of human typhoid fever and other infectious diseases. This provides researchers with an opportunity to study a poorly understood feature of human typhoid fever in a tractable animal model. Moreover, tissue culture hemophagocytosis assays will allow for the dissection of the molecular mechanisms by which the phagocytosed viable cells and/or the bacteria manipulate activated macrophages such that they become permissive for bacterial survival and replication.

## Materials and Methods

### Bacterial strains, growth conditions, and mouse infections.

Salmonella enterica serovar Typhimurium wild-type strain SL1344 [44] was grown overnight at 37 °C with aeration prior to infections. Antibiotics were used at the following concentrations: streptomycin, 30 μg/ml; kanamycin, 30 μg/ml; chloramphenicol, 20 μg/ml.

For mouse infections, 7-week-old female 129SvEvTac mice (Taconic Laboratories) were without food for 10–12 hours prior to intragastric inoculation with 5 × 10^8^ bacteria in 100uL of PBS.

### Processing of tissue sections for microscopy.

Liver and spleen samples were fixed in 4% paraformaldehyde, embedded in 2% agarose, and cut into 50 μm sections on a Leica Vibratome VT1000S. Sections were incubated in serum-free protein block (Dako Cytomation) containing 0.2% saponin and then with subsets of the following primary antibodies: rabbit anti-*S.* Typhimurium LPS O-antigen Group B polyclonal antisera (1:500; BD Biosciences), rat anti-mouse F4–80 and MOMA-2 (1:10; Serotec), phalloidin-Alexa488 (1:200; Molecular Probes), biotin-conjugated anti- Gr-1/Ly-6G/RB6-8C5 (1:25; MCA771B, Serotec), and biotin-conjugated hamster anti-CDɛ (1:25; BioLegend). Anti-Gr-1/Ly-6G/ RB6-8C5 recognizes a low-molecular-weight phosphatidylinositol-anchored cell surface glycoprotein expressed on granulocytes [12], a subset of eosinophils, plasmacytoid dendritic cells (which produce IFNα and IL-12 in response to viruses but not bacteria) [45], and transiently in the bone marrow during developmental stages of monocytes. This antibody does not cross-react with Ly-6C [46], as previously reported [47]. Sections were incubated with the following secondary antibodies: goat anti-rabbit-Alexa568, goat anti-rat-Alexa680, streptavidin-Alexa514, anti-hamster-Alexa546 (Molecular Probes). Sections were incubated with DAPI and mounted in ProLong Gold anti-fade reagent (Molecular Probes). Nick end-labeling was performed using Formalin-Fixed, Paraffin-Embedded (FFPE) tissues sectioned at 4 μm on a Leica Microtome RM2035, processed according to the Fluorescein FragEL DNA fragmentation Detection kit (Calbiochem) instructions, and counterstained with DAPI. Samples were analyzed on a Leica TCS SP2 Confocal Laser Scanning Microscope (CLSM) and processed with Image Analysis software. For activated caspase-3 labeling, FFPE 4-μm thin sections were stained with hematoxylin and eosin, incubated with rabbit anti-cleaved-caspase-3 antibody (Serotec) and anti-rabbit-Alexa568, and analyzed with light microscopy. Throughout experiments both liver and spleen were examined, but only liver images are shown because the regularly shaped hepatocytes facilitate visualization of irregularly shaped macrophages. Tissues from 4 mice at 1-week post-infection, and 2 mice at 3-, 4-, and 8-weeks post-infection were examined.

### Bone marrow–derived macrophage isolation and culture.

Bone marrow–derived macrophages (BMDMs) were isolated as previously described [48]. Briefly, marrow was flushed from femurs and humeri of 3.5–4.5 week old 129SvEvTac (Taconic) mice. Stem cells were isolated by overlaying on Histopaque-1083 (Sigma-Aldrich) and grown in Dulbecco modified Eagle medium (DMEM; Sigma-Alrich) supplemented w/ 10% heat-inactivated fetal bovine serum (FBS; HyClone), glutamine, sodium pyruvate, and 10 ng/ml granulocyte macrophage colony stimulating factor (GM-CSF; PeproTech) at 37 °C, 5% CO2 for 6 days [49,50]. Cells were assayed for expression of macrophage-specific markers, specifically a mixture of F4–80 and MOMA-2 as described above.

### Cell culture gentamicin protection assays.

BMDM or RAW264.7 cells (both are referred to as macrophages here, for clarity) were seeded at 10^5^ cells per well in poly-L-lysine-coated 24- or 96- well tissue culture plates. Cells were activated with 20 ng/ml lipopolysaccharide (S. *enterica* Typhimurium LPS; Sigma-Aldrich) and/or 20 U/ml IFNγ (PeproTech) for 18 hr and activation was measured with Griess assays. Activated and unactivated macrophages were incubated with media alone, polystyrene beads (2μm; Molecular Probes), or lymphocytes that were necrotic (30 min −80 °C), apoptotic (30 min at 56 °C [51,52]), or live. The lymphocytes included human T-cell-derived Jurkat E6–1 cells [53], human B-cell derived DG-75 cells [54], and mouse transgenic T-cells expressing RFP (Remi Creusot, Stanford University). Beads or cells were added to the macrophages at a ratio of 10:1 (beads/cells: macrophages). After 30 min (RAW264.7s) or 1 hr (BMDMs) macrophages were washed and infected for 30 min with normal mouse serum (Sigma) -opsonized *S.* Typhimurium at a multiplicity of infection of 20 (BMDMs) or 10 (RAW264.7s). Cells were washed and incubated for 1.5 hr at 37 °C in fresh media supplemented with gentamicin (100μg/ml) to kill extracellular bacteria. Media was exchanged for media supplemented with gentamicin (10 μg/ml) to prevent extracellular bacterial growth. At 2, 18 or 42 hr, wells were washed twice with pre-warmed PBS, incubated with 1% Triton X-100 for 5 min, lysed, and serial dilutions plated for colony-forming units. Percent survival was calculated by dividing CFUs obtained after 18 or 42 hr, by the initial number of intracellular bacteria after 2 hrs. Release of lactate dehydrogenase (LDH), a eukaryotic cytoplasmic enzyme, into the media correlates with cell death and was measured with the Cytotox-One kit (VWR) according to the kit instructions. There were not significant differences in BMDM cell death between samples or over the course of the experiments (data not shown). For immunofluorescence visualization of macrophages, the cytoplasm of Jurkat E6–1 or DG-75 cells was pre-labeled with CMFDA (Molecular Probes). Co-cultures were fixed with 4% paraformaldehyde and permeabilized with ice-cold methanol. Bacteria and/or macrophages were stained and visualized as described above. Statistical analyses were performed using a Students *t*-test.

## Supporting Information

Table S1 Macrophage and Dendritic Cell cDNAs That Change in Abundance upon Cell Exposure to Leishmania and Toxoplasma (104 KB DOC)

Video S1 Figure 1 Z-stack—4-μm Optical Sections through 1-wk-Infected Mouse Liver (81 MB AVI)

Video S2 Figure 2A–2C Z-stack—4-μm Optical Sections through 1-wk-Infected Mouse Liver (46 MB AVI)

Video S3 Confocal Rotation of T Cell (Pink) within a Macrophage (Blue), Actin Is Green (23 MB AVI)

### Accession Numbers

Derived from EntrezGene (http://www.ncbi.nlm.nih.gov/sites/entrez?db=gene).

Homo sapiens CD4/IAP/MER6/OA3 (961); Homo sapiens Leukocyte Specific Transcript 1 LST1 (7940); Homo sapiens Major Histocompatibility Complex, Class II, DM Beta HLA-DMB (3109); Homo sapiens Signal-Regulatory Protein Alpha /SIRPalpha/SHPS-1 (140885); Homo sapiens Syntaxin 11/ STX11 (8676); Homo sapiens V-Ets Erythroblastosis Virus E26 Oncogene Homolog 2 (avian)/ETS2 (2114); Mus musculus CD3/ Cd247/ 4930549J05Rik/ AW552088/ CD3-eta/ CD3-zeta/ Cd3h/ Cd3z/ T3z/ TCRk/ Tcrz (12503); Mus musculus F4–80/ Cell Surface Glycoprotein F4/80; Lymphocyte Antigen 71/ DD7A5–7/ EGF-TM7/ F4/80/ Gpf480/ Ly71/ TM7LN3 (13733); Mus musculus Lymphocyte Antigen 6 Complex, Locus G/ Ly-6G/Gr-1/Gr1/Ly6g (17072); Mus musculus Slc11a1/Nramp1 (18173).
