# Supplementary material for: Hemophagocytic Macrophages Harbor Salmonella enterica during Persistent Infection
Source: PLoS Pathog. 2007 Dec 14;3(12):e193. doi: 10.1371/journal.ppat.0030193 (PMC2134957; doi:10.1371/journal.ppat.0030193)
Supplement: Supplementary file 1 [file ppat.0030193.st001.doc]

| **Supplementary Table 1**  **Macrophage and dendritic cell cDNAs that change in abundance upon cell exposure to Leishmania and Toxoplasma^1^** | | | | |
| --- | --- | --- | --- | --- |
| **Gene Name** | **gb accession** | **Biological function^2^** | **PMID** | **Fold difference^3^** |
| **More highly expressed in cells incubated with parasites** |  |  |  |  |
| G0S2, G0/G1switch 2 | NM_015714 | Lymphocyte G0/G1 switch | 16086669 | 7.4 |
| ETS2, v-ets erythroblastosis virus E26 oncogene homolog 2 (avian) | J04102 | Transcription factor, activates IL-12 p40 | 16518544 | 7.1 |
| GADD45B, growth arrest and DNA-damage-inducible, beta | NM_015675 | MAPKKK activator, myeloid stress response | 9827804 | 4.6 |
| STX11, syntaxin 11 | NM_003764 | Vesicle trafficking; mutations associated with primary hemophagocytic lymphohistiocytosis | 16278825 | 4.3 |
| CFLAR, CASP8 and FADD-like apoptosis regulator | AF005775 | Death effector domain-containing protein; interacts with caspase-8 and regulates apoptosis | 9380701 | 3.7 |
| MT2A, metallothionein 2A | NM_005953 | metal ion binding, protective against ROS damage | 16402917 | 3.7 |
| VEGFA, vascular endothelial growth factor A | AF024710 | Role in vascular development | 17698971 | 3.3 |
| BTG3, BTG family, member 3 | NM_006806 | Negative regulator of the cell cycle | 9067576 | 3.2 |
| BTG1, B-cell translocation gene 1, anti-proliferative | NM_001731 | Growth arrest | 15033446 | 3.0 |
| WTAP, Wilms tumor 1 associated protein | NM_004906 | Regulates the proliferation of vascular smooth muscle cells | 17095724 | 3.0 |
| NRP2, neuropilin 2 | NM_003872 | Interacts with VEGFR-2 and VEGFR-3 and promotes endothelial cell survival and migration | 16621967 | 2.8 |
| AK3L1, adenylate kinase 3-like 1 | NM_001005353 | Localized to the mitochondrial matrix | 11485571 | 2.8 |
| RAPGEF2, Rap guanine nucleotide exchange factor (GEF) 2 | NM_014247 | Activates Rap1 and Rap2, which regulate the actin cytoskeleton | 17382517 | 2.6 |
| DPT, dermatopontin | NM_001937 | Extracellular matrix protein, enhances TGFbeta activity | 9895299 | 2.6 |
|  |  |  |  |  |
| **More highly expressed in cells incubated without parasites** |  |  |  |  |
| P2RY14, purinergic receptor P2Y, G-protein coupled, 14 | NM_014879 | Functions with P2Y receptors | 16109883 | -7.0 |
| FCN1, ficolin (collagen/fibrinogen domain containing) 1 | NM_002003 | Plasma protein expressed by peripheral blood leukocytes, activates complement | 16305643 | -5.8 |
| CORO1A, coronin, actin binding protein, 1A | NM_007074 | Required for chemotaxis and phagocytosis in neutrophils | 17442961 | -5.1 |
| ADORA3, adenosine A3 receptor | NM_000677 | A3 and P2Y2 receptors guide neutrophil chemotaxis upon ATP release | 17170310 | -4.9 |
| CAMK1, calcium/calmodulin-dependent protein kinase I | L41816 | Signaling | 7641687 | -4.6 |
| S100A4, S100 calcium binding protein A4 | NM_002961 | Role in apoptosis inhibition | 17638890 | -4.1 |
| AIF1, allograft inflammatory factor 1 | NM_001623 | IFNgamma induced, negative regulated growth of vascular smooth muscle cells | 17498268 | -3.9 |
| ETV5, ets variant gene 5 (ets-related molecule) | NM_004454 | Transcription factor, activates matrix metalloproteinase-2 (MMP-2) | 17638886 | -3.8 |
| FGL2, fibrinogen-like 2 | NM_006682 | Secreted mucosal protein | 16437596 | -3.7 |
| NDUFB7, NADH dehydrogenase (ubiquinone) 1 beta subcomplex, 7, 18kDa | NM_004146 |  | 11864782 | -3.7 |
| METTL7A, methyltransferase like 7A | NM_014033 |  |  | -3.6 |
| STAB1, stabilin 1 | NM_015136 | Internalizes and targets SPARC (Secreted Proteins Acidic and Rich in Cysteine) to endosomal pathway | 16670288 | -3.6 |
| LST1, leukocyte specific transcript 1 | NM_007161 | Targets the ERK/MAP kinase scaffold proteins KSR, MEK1, and ERK2 to the actin cytoskeleton | 15090600 | -3.4 |
| CPSF1, cleavage and polyadenylation specific factor 1, 160kDa | NM_013291 | Subunit of CPSF that binds to U-rich RNA elements and stimulates poly(A) polymerase | 14749727 | -3.4 |
| GAS7, growth arrest-specific 7 | NM_003644 | Association with F-actin induces reorganization of microfilaments and promotes membrane outgrowth | 11795944 | -3.3 |
| FCER1A, Fc fragment of IgE, high affinity I, receptor for; alpha polypeptide | NM_002001 | On mast cells, binds IgE | 17267694 | -3.2 |
| TRIM44, tripartite motif-containing 44 | NM_017583 | The TRIM motif includes three zinc-binding domains | 11331580 | -3.2 |
| SULT1A2, sulfotransferase family, cytosolic, 1A, phenol-preferring, member 2 | U34804 | metabolizes aromatic amines | 16399348 | -3.1 |
| PPM1F, protein phosphatase 1F (PP2C domain containing) | NM_014634 | Plays key role in Ca2+/calmodulin-dependent protein kinase II (CaMKII) regulation | 15140879 | -3.1 |
| CPVL, carboxypeptidase, vitellogenic-like | NM_019029 | Localizes to ER and membrane ruffles in macrophages | 16436111 | -3.0 |
| FES, feline sarcoma oncogene | X06292 |  |  | -3.0 |
| HLA-DMB, major histocompatibility complex, class II, DM beta | NM_002118 | MHCII peptide loading; antigen presenting cell activation marker | 12932352 | -3.0 |
| C11orf51, chromosome 11 open reading frame 51 | NM_014042 |  | 10602999 | -3.0 |
| PECAM1, platelet/endothelial cell adhesion molecule (CD31 antigen) | NM_000442 | Can confer resistance to apoptosis | 17106245 | -2.8 |
| PTPN7, protein tyrosine phosphatase, non-receptor type 7 | NM_002832 | MAPK-specific protein tyrosine phosphatase, overexpressed in myeloid malignancies | 16441242 | -2.7 |
| INPP5D, inositol polyphosphate-5-phosphatase, 145kDa | U57650 | SH2-containing inositol 5-phosphatase (hSHIP), associates with SHC after cytokine stimulation | 8874179 | -2.7 |
| NCF4, neutrophil cytosolic factor 4, 40kDa, p40phox | NM_000631 | NADPH oxidase subunit, binds phosphoinositide during FcgammaIIA receptor-induced phagocytosis | 16880255 | -2.7 |
| TSPAN4, tetraspanin 4, gb=AF022813, gi=2586349 | AF022813 | Transmembrane-4 superfamily (TM4SF) protein that complexes with integrins and other TM4SF proteins | 9360996 | -2.7 |
| ALDH5A1, aldehyde dehydrogenase 5 family, member A1 (succinate-semialdehyde dehydrogenase) | NM_001080 | Higher activity is associated with higher intelligence across the general population | 14635103 | -2.7 |
| BLVRB, biliverdin reductase B (flavin reductase (NADPH)) | NM_000713 |  | 7656592 | -2.7 |
| LMO2, LIM domain only 2 (rhombotin-like 1) | NM_005574 | Interacts with Bex2 to regulate transcription in B-cells and myleoid precursors | 17038524 | -2.6 |
| ADCY7, adenylate cyclase 7 | NM_001114 | Signaling | 9034014 | -2.6 |
| CXorf9, chromosome X open reading frame 9 | NM_018990 | Novel SH3 protein (SLY) preferentially expressed in lymphoid cells | 11470164 | -2.6 |
| CLEC3B, C-type lectin domain family 3, member B | NM_003278 | Plasminogen kringle 4-binding protein | 1354271 | -2.6 |
| FUCA1, fucosidase, alpha-L- 1, tissue | NM_000147 | Recessive mutations lead to a lysosomal storage disease and fucose accumulation in tissues | 12408193 | -2.6 |
| SPTBN1, spectrin, beta, non-erythrocytic 1 | NM_003128 | Functional partner of ankyrin-G in the biogenesis of lateral membrane of epithelial cells | 17074766 | -2.5 |
| AOAH, acyloxyacyl hydrolase (neutrophil) | NM_001637 | Deacylates bacterial lipopolysaccharides | 1987068 | -2.5 |
| GMFG, glia maturation factor, gamma | NM_004877 | Hematopoietic-specific, may mediate the pluripotentiality and lineage commitment of stem cells | 17127212 | -2.5 |
| BTK, Bruton agammaglobulinemia tyrosine kinase | NM_000061 | Phosphorylates Mal (MyD88 adapter-like) during TLR2 and TLR4 signal transduction | 16439361 | -2.5 |
| PRCP, prolylcarboxypeptidase (angiotensinase C) | NM_005040 | Lysosomal prolylcarboxypeptidase, cleaves angiotensions, bradykinins, and prekallikrein | 14996700 | -2.5 |
| PLCB2, phospholipase C, beta 2 | M95678 | Signaling | 15274049 | -2.5 |
|  |  |  |  |  |
| 1. Data derived from NCBI GEO Database, Series GSE 360, PMID: 12663451 | | |  |  |
| 2. Blue text indicates gene function is relevant to macrophage or dendritic cell activation state | | |  |  |
| 3. Average infected cDNA level divided by average uninfected cDNA level. Negative values indicate that cDNA levels were lower in infected samples. Genes shown had cDNA levels that differed by at least 2.5 fold, *P*≤0.001. | | |  |  |
